# Glutathione Ethyl Ester Protects In Vitro*-*Maturing Bovine Oocytes against Oxidative Stress Induced by Subsequent Vitrification/Warming

**DOI:** 10.3390/ijms21207547

**Published:** 2020-10-13

**Authors:** Tania García-Martínez, Meritxell Vendrell-Flotats, Iris Martínez-Rodero, Erika Alina Ordóñez-León, Manuel Álvarez-Rodríguez, Manel López-Béjar, Marc Yeste, Teresa Mogas

**Affiliations:** 1 Department of Animal Medicine and Surgery, Autonomous University of Barcelona, ES-08193 Cerdanyola del Vallès, Spain; taniagarciamartinez@gmail.com (T.G.-M.); meritxell.vflotats@gmail.com (M.V.-F.); iris.martinez@outlook.com (I.M.-R.); alina.mvzalina@gmail.com (E.A.O.-L.); 2 Department of Animal Health and Anatomy, Autonomous University of Barcelona, ES-08193 Cerdanyola del Vallès, Spain; Manuel.Alvarez.Rodriguez@uab.cat (M.Á.-R.); Manel.Lopez.Bejar@uab.cat (M.L.-B.); 3 Grupo InVitro, Tabasco 86040, Mexico; 4 College of Veterinary Medicine, Western University of Health Sciences, Pomona, CA 91766, USA; 5 Department of Biology, Institute of Food and Agricultural Technology, University of Girona, ES-17004 Girona, Spain; marc.yeste@udg.edu

**Keywords:** cow, reactive oxygen species, cryopreservation, cryotop, spindle configuration, mitochondria, embryo development, gene expression

## Abstract

This study aimed to examine whether the addition of glutathione ethyl ester (GSH-OEt) to the in vitro maturation (IVM) medium would improve the resilience of bovine oocytes to withstand vitrification. The effects of GSH-OEt on spindle morphology, levels of reactive oxygen species (ROS), mitochondrial activity and distribution, and embryo developmental potential were assessed together with the expression of genes with a role in apoptosis (*BAX, BCL2*), oxidative-stress pathways (*GPX1, SOD1*), water channels (*AQP3*), implantation (*IFN-τ*) and gap junctions (*CX43*) in oocytes and their derived blastocysts. Vitrification gave rise to abnormal spindle microtubule configurations and elevated ROS levels. Supplementation of IVM medium with GSH-OEt before vitrification preserved mitochondrial distribution pattern and diminished both cytoplasmic and mitochondrial ROS contents and percentages of embryos developing beyond the 8-cell stage were similar to those recorded in fresh non-vitrified oocytes. Although not significantly different from control vitrified oocytes, vitrified oocytes after GSH-OEt treatment gave rise to similar day 8-blastocyst and hatching rates to fresh non-vitrified oocytes. No effects of GSH-OEt supplementation were noted on the targeted gene expression of oocytes and derived blastocysts, with the exception of *GPX1*, *AQP3* and *CX43* in derived blastocysts. The addition of GSH-OEt to the IVM medium before vitrification may be beneficial for embryo development presumably as the consequence of additional anti-oxidant protection during IVM.

## 1. Introduction

The successful cryopreservation of mammalian oocytes is useful for assisted reproductive technologies including animal breeding programs and somatic cell nuclear transfer as this method of storage resolves the temporal and spatial limitations of oocyte supplies (for a review, see [1,2]). Vitrification has proved more efficient and reliable than slow freezing for the cryopreservation of oocytes of many species including cow [3,4,5], mouse [6], and human [7,8], among others. However, despite the increasing interest in this approach and its applications, yields of transferable blastocysts after the in vitro fertilization of vitrified bovine oocytes remain low [1,9].

The impaired embryo developmental potential of vitrified oocytes has been attributed to abnormal meiotic spindle assembly, destabilization of the microfilament and microtubular elements of the cytoskeleton, plasma membrane rupture, premature cortical granule exocytosis, and zona pellucida hardening, among other factors (reviewed by [1]). Moreover, vitrification severely affects the morphofunctional integrity of the oocyte’s mitochondria and endogenous antioxidant systems, with the consequence of increased levels of reactive oxygen species (ROS) [10,11]. The regulation of intracellular redox potential is a crucial determinant of oocyte competence [12]. If the increased levels of ROS cannot be eliminated, ROS can cause meiotic spindle disassembly and chromosome misalignment, mitochondrial damage and ATP depletion [13,14]. Further, ROS can compromise the developmental capacity of embryos and promote apoptosis in oocytes and early embryos [14,15] by activating the caspase cascade that executes the apoptotic program [16,17].

Glutathione (GSH), a tripeptide thiol (γ-glutamylcysteinylglycine), is the major non-enzymatic line of defense against oxidative stress owing to the reducing power of its sulfhydryl group [18]. In effect, intracellular GSH synthesis is a critical part of oocyte cytoplasm maturation and relies on the γ-glutamyl cycle during oocyte maturation. In hamster oocytes, GSH concentrations rise during germinal vesicle breakdown, peak at metaphase II and rapidly decline in zygotes and embryos at the early developmental stage [19]. Appropriate GSH reservoirs attained during in vitro maturation (IVM) are essential for the formation, maintenance and protection of the meiotic spindle against oxidative stress, the formation of the male pronucleus after fertilization [20] and further embryo development [21]. Oocytes and early embryos have a limited capacity to synthesize GSH until they develop to the blastocyst stage. Besides, extracellular GSH cannot penetrate the cell membrane or enter the oocytes/embryos before this blastocyst stage. Cumulus cells surrounding oocytes play a crucial role in intracellular GSH synthesis by absorbing thiols such as β-mercaptoethanol and cysteine through gap junctions, which are then synthesized to GSH through the γ-glutamyl cycle [22]. In fact, the addition of such thiol compounds to the IVM medium can increase intracellular GSH levels and improve the developmental potential of these oocytes in several domestic species including pigs [23] and cattle [24]. However, when cumulus cells are removed during vitrification, this limits the capacity of the oocytes to synthesize GSH, leading to a low fertilization rate and reduced developmental potential [25]. Thus, although vitrified bovine oocytes can be stimulated by IVM treatment with β-mercaptoethanol/cysteine to produce high intracellular GSH levels, this high GSH content was not found to improve a high incidence of multiple aster formation and the poor potential of fertilized oocytes to develop to the blastocyst stage [26]. This could indicate that the addition of these small thiols to the IVM medium prior to cryopreservation failed to produce sufficient amounts of GSH to overcome the negative impacts of vitrification on oocyte metabolism.

Curnow et al. [27,28] showed that 5 mM of glutathione ethyl ester (GSH-OEt), a novel oocyte-permeable, cumulus cell-independent GSH donor, is able to enhance oocyte GSH levels during maturation, improving rates of maturation, normal spindle alignment and fertilization in in vitro matured bovine and primate oocytes. GSH-OEt is a small fat-soluble molecule, which penetrates cell membranes efficiently and is hydrolyzed to GSH by intracellular esterases. It does not, therefore, rely on the γ-glutamyl cycle or require energy-dependent transport across cell membranes [29]. When added to the IVM medium, GSH-OEt has been observed to improve mitochondrial functionality and regulation of redox homeostasis in vitrified/warmed IVM murine oocytes and has been also related to a more rapid recovery of spindle birefringence and improved further embryo development [30].

The present study was thus designed to examine the efficacy of 5 mM GSH-OEt added to the in vitro maturation medium prior to vitrification/warming in terms of its capacity to reduce oxidative stress and enhance the developmental competence of vitrified bovine oocytes after their in vitro fertilization. In MII oocytes and blastocysts derived from oocytes supplemented with GSH-OEt, we also assessed the relative mRNA abundances of genes involved in apoptosis, oxidative-stress pathways, water channels, implantation and gap junctions.

## 2. Results

### 2.1. Meiotic Spindle Status of Vitrified/Warmed Bovine Oocytes after Maturation in IVM Medium Supplemented with GSH-OEt

**Figure 1:**
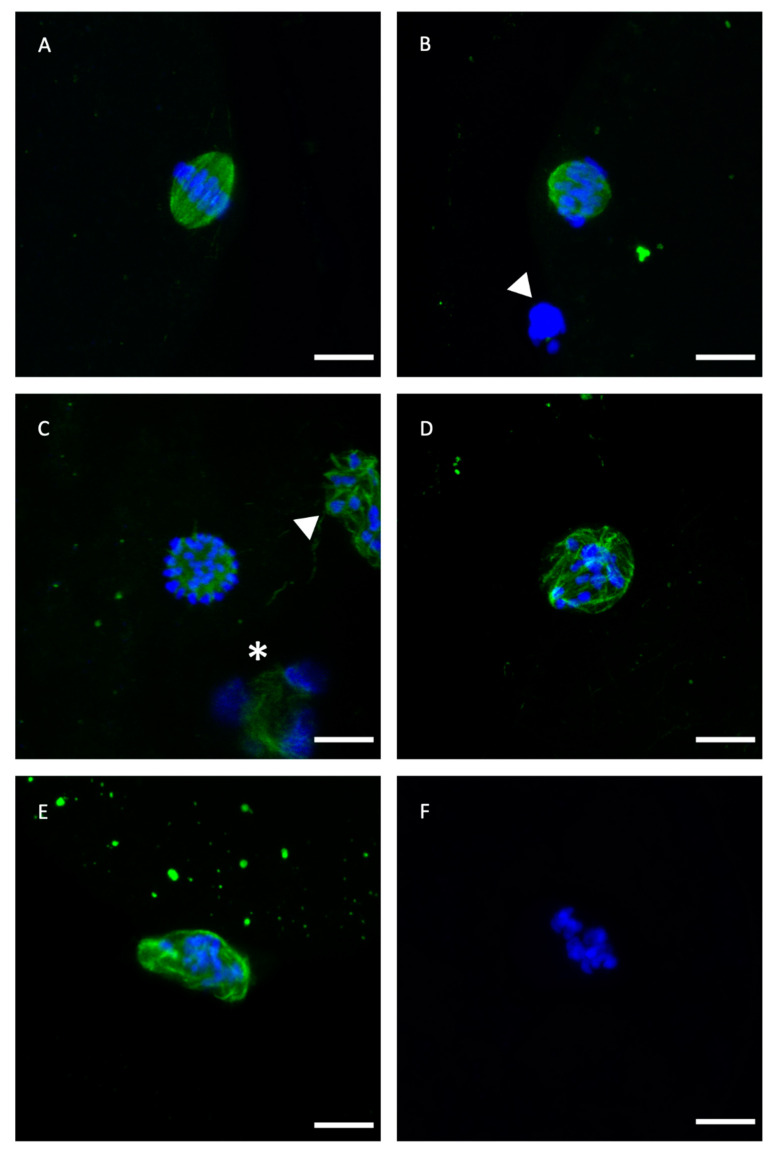
Representative confocal laser-scanning photomicrographs of spindle and chromosome configurations of IVM bovine oocytes after vitrification with or without GSH-OEt pretreatment. (**A**) Normal barrel-shaped MII spindle with microtubules forming a clear meiotic spindle with chromosomes aligned at its equator. (**B**,**C**) Abnormal spindle morphology showing partly disorganised chromosomes. (**D**) Abnormal spindle structure associated with a disrupted microtubule arrangement and chromosomes appearing condensed. (**E**) Disrupted microtubule shape. Note the decondensation of microtubules and the less condensed appearance of chromosomes. (**F**) Single block of condensed chromatin in the absence of microtubules. Scale bar = 10 µm. Green, tubulin (Alexa Fluor™ 488); blue, chromosomes (DAPI). The white arrowhead indicates polar body and white asterisk indicates cumulus cell nuclei.

Table 1 compares the effects of adding or not 5 mM GSH-OEt to the IVM medium prior to vitrification on oocyte spindle and chromosome organization. No significant differences in percentages of metaphase II or normal spindle configuration were observed between treatments. Thus, rates of oocytes showing an abnormal microtubule configuration were similar in fresh control oocytes and VIT GSH-OEt, although no differences were observed among VIT GSH-OEt, VIT control and GSH-OEt groups. However, vitrified control oocytes showed significantly higher percentages of abnormal microtubule configurations when compared to fresh control oocytes. Detailed images of these normal and abnormal patterns are provided in Figure 1.

### 2.2. ROS Production Detected in Vitrified/Warmed Bovine Oocytes after Their Maturation in IVM with or without GSH-OEt

**Figure 2:**
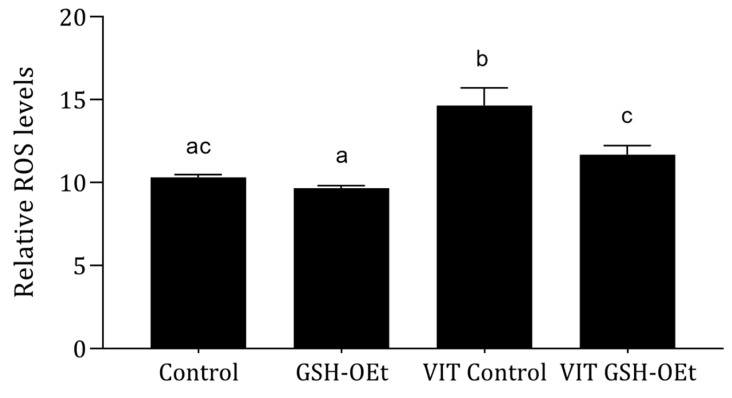
Effect of adding 5 mM GSH-OEt to the IVM medium before vitrification on relative reactive oxygen species (ROS) fluorescence intensity in bovine oocytes. ^a,b,c^ Different superscript letters within columns indicate significant differences (*p* < 0.05). Data are shown as mean ± SEM. Treatment groups: *Control*, oocytes in vitro matured in IVM medium; *GSH-OEt*, oocytes in vitro matured in IVM medium supplemented with 5 mM GSH-OEt; *VIT Control*, oocytes in vitro matured in IVM medium and then vitrified on Cryotops followed by warming; *VIT GSH-OEt*, oocytes in vitro matured in IVM medium supplemented with 5 mM of GSH-OEt and then vitrified on Cryotops followed by warming.

Relative ROS levels measured in MII oocytes matured with or without GSH-OEt supplementation prior to vitrification/warming are shown in Figure 2. Vitrification led to significantly higher levels of ROS (*n =* 38; 14.64 ± 1.07) (*p* < 0.05) when compared to the other treatments. However, oocytes vitrified after maturation in IVM medium supplemented with 5 mM GSH-OEt (VIT GSH-OEt group *n =* 40; 11.68 ± 0.55) showed similar ROS levels to control non-vitrified oocytes matured in the absence of GSH-OEt (Control group *n =* 65; 10.30 ± 0.18).

### 2.3. Mitochondrial Activity and Distribution in Vitrified/Warmed Bovine Oocytes after Their Maturation in IVM Medium Supplemented with GSH-OEt

**Figure 3:**
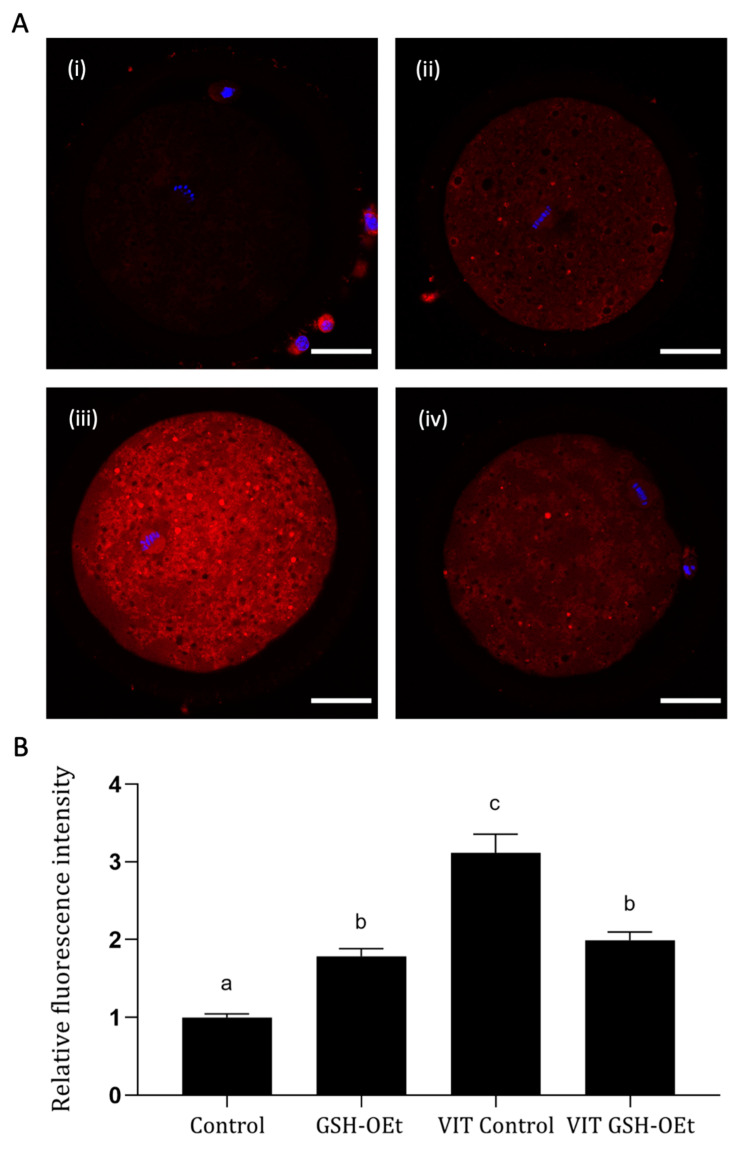
Effect of the addition of 5 mM GSH-OEt to the IVM medium before vitrification on mitochondrial oxidative activity in bovine oocytes. Data are shown as mean + SEM. ^a,b,c^ Different superscripts within columns indicate significant differences (*p* < 0.05). Representative images of MII oocytes stained with MitoTracker^®^ Red CM-H_2_XRos (red) and Hoechst nuclear staining (blue): (**A**) (i) Control, (ii) GSH-OEt, (iii) VIT Control, (iv) VIT GSH-OEt. Scale bar = 30 µm. (**B**) Relative fluorescence intensity of mitochondrial oxidative activity in oocytes. Treatment groups: *Control*, oocytes in vitro matured in IVM medium; *GSH-OEt*, oocytes in vitro matured in IVM medium supplemented with 5 mM GSH-OEt; *VIT Control*, oocytes in vitro matured in IVM medium and then vitrified on Cryotops followed by warming; *VIT GSH-OEt*, oocytes in vitro matured in IVM medium supplemented with 5 mM of GSH-OEt and then vitrified on Cryotops followed by warming.

We examined the effects of GHS-OEt supplementation prior to vitrification/warming on mitochondrial distribution in MII oocytes by labelling them with a cell-permeable MitoTracker^®^ Red CM-H_2_XRos. Detailed images of fluorescence labelling are shown in Figure 3A. As shown in Figure 3B, vitrification significantly increased mitochondrial oxidative activity in the MII oocytes after IVM (*n* = 48, 3.12 ± 0.24) when compared to other treatments. While significantly lower mitochondrial oxidative activity was observed in fresh control oocytes (*n* = 44, 1.00 ± 0.05), similar mitochondrial oxidative activity was observed between non-vitrified GSH-OEt (*n* = 45, 1.79 ± 0.10) and vitrified GSH-OEt oocytes (*n* = 44, 1.99 ± 0.11).

**Figure 4:**
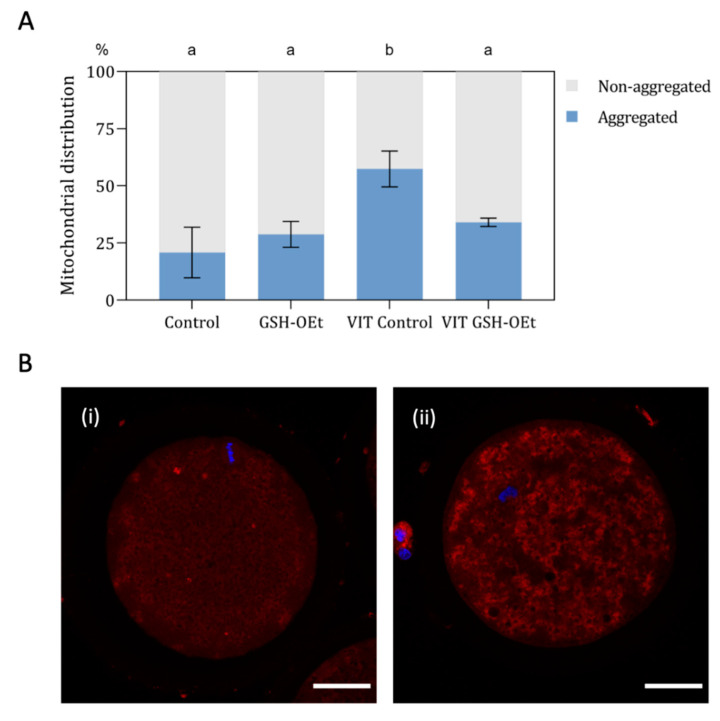
Effect of the addition of 5 mM GSH-OEt to the IVM medium before vitrification on mitochondrial distribution in bovine oocytes. Data are shown as mean + SEM. ^a,b^ Different superscripts within columns indicate significant differences (*p* < 0.05). (**A**) Distribution of MII oocytes according to mitochondrial distribution pattern: (i) non-aggregated mitochondrial distribution; (ii) aggregated mitochondrial distribution. (**B**) Representative images of mitochondrial distribution with MitoTracker^®^ Red CM-H_2_XRos (red) and Hoechst nuclear staining (blue). Mitochondrial distribution was categorized according to the presence or absence of two or more aggregates in the oocyte cytoplasm into (i) non-aggregated or (ii) aggregated. Scale bar = 30 µm. Treatment groups: *Control*, oocytes in vitro matured in IVM medium; *GSH-OEt*, oocytes in vitro matured in IVM medium supplemented with 5 mM GSH-OEt; *VIT Control*, oocytes in vitro matured in IVM medium and then vitrified on Cryotops followed by warming; *VIT GSH-OEt*, oocytes in vitro matured in IVM medium supplemented with 5 mM of GSH-OEt and then vitrified on Cryotops followed by warming.

When mitochondrial distribution was assessed (Figure 4A), a significantly higher percentage of MII oocytes showing a mitochondrial aggregate pattern was observed in the vitrified group (*n* = 48, 57.40% ± 7.81) when compared to other treatments (Control: *n* = 44; 20.80% ± 11.02; GSG-OEt: *n* = 45; 28.80% ± 5.69 and VIT GSH-OEt: *n* = 44; 34.00% ± 1.84. Figure 4B shows the details of mitochondrial distribution patterns.

### 2.4. Gene Expression in Vitrified/Warmed Bovine Oocytes after Their Maturation in IVM Medium Supplemented with GSH-OEt

**Figure 5:**
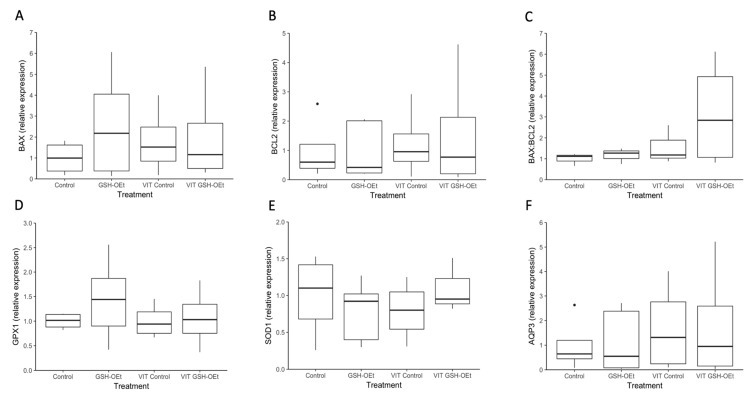
Box-and-whisker plot showing relative gene expression levels of (**A**) *BAX*, (**B**) *BCL2*, (**C**) *BAX:BCL2*, (**D**) *GPX1*, (**E**) *SOD1* and (**F**) *AQP3* in MII bovine oocytes in vitro matured in IVM medium with or without GSH-OEt before their vitrification. Box compartments represent 25th and 75th percentiles and whiskers represent maximum and minimum values. The line across the boxes represents the median. *BAX*, BCL2 associated X, apoptosis regulator; *BCL2*, BCL2 apoptosis regulator; *GPX1*, glutathione peroxidase 1; *SOD1*, superoxide dismutase 1; *AQP3*, aquaporin 3. *Control*, oocytes in vitro matured in IVM medium; *GSH-OEt*, oocytes in vitro matured in IVM medium supplemented with 5 mM GSH-OEt; *VIT Control*, oocytes in vitro matured in IVM medium and then vitrified on Cryotops followed by warming; *VIT GSH-OEt*, oocytes in vitro matured in IVM medium supplemented with 5 mM of GSH-OEt and then vitrified on Cryotops followed by warming.

Data on the relative abundances of mRNA transcripts in MII oocytes in vitro matured with GSH-OEt before vitrification/warming are provided in Figure 5. No differences were observed in relative abundances of transcripts of genes related to oxidative-stress (*GPX1, SOD1)*, water channels (*AQP3*) and apoptosis (*BAX, BCL2)* as well as in the *BAX:BCL2* ratio in MII oocytes, regardless of treatment.

### 2.5. Developmental Competence and Gene Expression Determined in Embryos Derived from Vitrified/Warmed Bovine Oocytes In Vitro Matured in IVM Medium with or without GSH-OEt

The effects observed of GSH-OEt supplementation during in vitro maturation before vitrification/warming on embryo development are detailed in Table 2. Matured non-vitrified oocytes gave rise to a significantly higher cleavage rate than vitrified oocytes, regardless of GSH-OEt treatment. In vitro maturation with GSH-OEt led to significantly higher D7 blastocyst rates than those recorded in both groups of vitrified oocytes but similar to values observed for non-vitrified non-GSH-OEt treated oocytes. Embryo development up to the 16-cell or blastocyst stage was lower for oocytes vitrified without prior GSH-OEt treatment compared to non-vitrified oocytes. However, oocytes vitrified after IVM with GSH-OEt yielded similar 16-cell stage and D8 blastocyst rates than non-vitrified oocytes. Likewise, while similar hatching D8-blastocyst rates were observed in the oocytes not vitrified and those vitrified after GSH-OEt treatment, D8 blastocysts derived from non-GSH-OEt-treated vitrified oocytes showed a significantly lower hatching capacity than oocytes in the non-vitrified groups.

**Figure 6:**
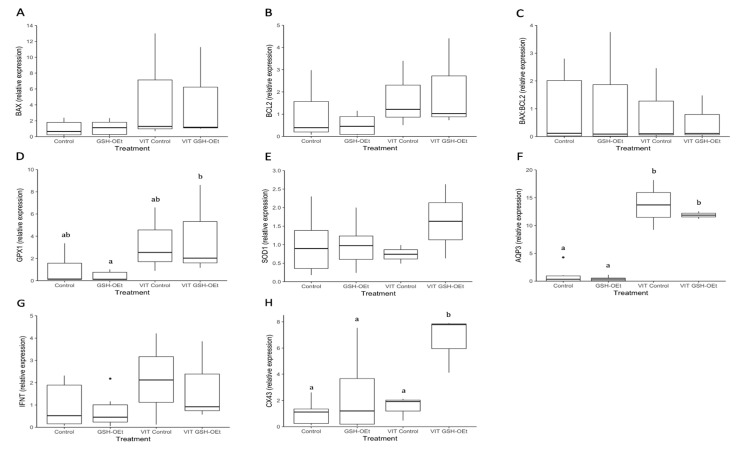
Box-and-whisker plot showing relative gene expression levels of (**A**) *BAX*, (**B**) *BCL2*, (**C**) *BAX:BCL2* ratio, (**D**) *GPX1*, (**E**) *SOD1*, (**F**) *AQP3*, (**G**) *IFN-τ* and (**H**) *CX43* in D8 bovine blastocysts derived from oocytes in vitro matured in IVM medium with or without GSH-OEt before their vitrification. Box compartments represent 25th and 75th percentiles and whiskers represent maximum and minimum values. The line across the boxes represents the median. ^a,b^ Different letters indicate statistically significant differences (*p < 0.05*). *BAX*, BCL2 associated X, apoptosis regulator; *BCL2*, BCL2 apoptosis regulator; *GPX1*, glutathione peroxidase 1; *SOD1*, superoxide dismutase 1; *AQP3*, aquaporin 3; *IFN-τ*, interferon-tau-c2; *CX43*, connexin 43. *Control*, oocytes in vitro matured in IVM medium; *GSH-OEt*, oocytes in vitro matured in IVM medium supplemented with 5 mM GSH-OEt; *VIT Control*, oocytes in vitro matured in IVM medium and then vitrified on Cryotops followed by warming; *VIT GSH-OEt*, oocytes in vitro matured in IVM medium supplemented with 5 mM of GSH-OEt and then vitrified on Cryotops followed by warming.

Through RT-qPCR, we obtained an overview of the expression levels of seven genes in D8-blastocysts derived from oocytes vitrified after GSH-OEt treatment (Figure 6). No significant differences in the relative abundances of genes involved in apoptosis (*BAX, BCL2)*, oxidative-stress (*SOD1*) and implantation (*IFN-τ*) were observed in D8-blastocysts derived from fresh or vitrified/warmed oocytes, regardless of GSH-OEt treatment (Figure 6A,B,E,G, respectively). Although the abundance of *GPX1* transcripts observed in blastocysts derived from vitrified oocytes previously matured with GSH-OEt did not differ from levels observed in blastocysts derived from vitrified or fresh non-treated oocytes, blastocysts in the VIT GSH-OEt group showed significantly higher levels of *GPX1* transcripts compared to those in the non-vitrified GSH-OEt group (Figure 6D). Blastocysts derived from vitrified/warmed oocytes showed significantly (*p* < 0.05) higher relative *AQP3*-transcript abundances than blastocysts derived from fresh oocytes, regardless of GSH-OEt supplementation during in vitro maturation (Figure 6F). Expression levels of *CX43* gene transcripts were significantly (*p < 0.05*) higher in D8 blastocysts arising from vitrified/warmed oocytes matured with GSH-OEt when compared to blastocysts derived from oocytes in the non-treated vitrified group or both non-vitrified groups (Figure 6H).

## 3. Discussion

The cryopreservation of bovine MII stage oocytes by vitrification induces heat stress and osmotic stress-causing critical damage to cellular organelles [1,9]. GSH plays an important role in maintaining a balanced intracellular redox state to protect cells against the harmful effects of oxidative stress [31]. In the present study, GSH-OEt was added as a supplement to the medium during the IVM of bovine oocytes to examine its protective role against damage induced by the vitrification/warming protocol.

The process of spindle formation and chromosome segregation is particularly sensitive to both the physical and chemical environment [32]. The correct configuration of these structures is essential in the events following oocyte fertilization such as meiosis completion, second polar body formation, pronuclei migration, and first mitotic spindle formation [33]. Disorganization of the meiotic spindles could result in chromosome dispersion, failure of normal fertilization, and end of development [34]. Increased ROS levels arising from vitrification can attack microtubules and interfere with spindle formation [13]. In compensation, increased levels of GSH support the correct assembly of microtubules by preventing ROS from attacking tubulin assembly, and maintain normal spindle function during meiosis [35,36]. When we examined spindle and chromosome behavior by tubulin immunofluorescence in the present study, we found no differences in percentages of normal-shaped spindles in vitrified and non-vitrified oocytes, regardless of GSH-OEt treatment. Trapphoff et al. [30] observed that oocytes vitrified after pre-incubation with GSH-OEt had normal spindles, and while abnormalities were not significantly different from those detected in fresh controls, they were significantly lower compared to abnormalities observed directly after warming in vitrified oocytes. However, irrespective of GSH-OEt supplementation, these authors noted that spindle and chromosome configurations were re-established 2 h after vitrification [30]. This post-warming interval of 2 h at 37 °C was likely sufficient to restore spindle structure regardless of treatment, and could mask the beneficial effects of GSH-OEt pretreatment on spindle morphology. Contrarily, Li et al. [37] observed that GSH-OEt preincubation improved spindle morphology after the vitrification of in vivo matured mouse oocytes. Our study revealed that vitrification significantly increases the percentage of oocytes displaying abnormal microtubule configurations, as reported in previous studies carried out by our group [38]. However, no differences in abnormal microtubule configuration were observed in oocytes vitrified after IVM with GSH-OEt when compared to non-vitrified oocytes. We surmise the spindle may be particularly protected by GSH-OEt, as high levels of this thiol may prevent oxidation of cysteine sulphydryl-groups of the αß tubulin dimers in microtubules [35,36] and avoid the oxidation of cellular structures within mitochondria, which are affected by vitrification.

Vitrification induces damage to the endogenous antioxidant systems of oocytes, with consequent increases in ROS activity produced when metabolism resumes and cytoplasmic damage has been repaired [39]. In effect, it has been established that ROS levels rise after oocyte vitrification [10,14,37,40]. When ROS levels were assessed in our study, pretreatment with GSH-OEt prior to vitrification reduced ROS contents to similar levels to those of oocytes in the control non-vitrified group, while ROS levels in vitrified non-treated oocytes were significantly higher. Similarly, GSH-OEt preincubation improved mitochondrial distribution and reduced levels of intracytoplasmic ROS in vitrified mouse oocytes leading to improved embryo development and underlying the beneficial effect of GSH-OEt incubation prior to vitrification [37].

Mitochondria play a key role in adenosine triphosphate (ATP) generation for oocyte and embryonic development [41]. It is well known that mitochondria distribution is a dynamic process, and it is an important indicator of oocyte quality [42]. For example, a uniform, granulated distribution of active mitochondria in the process of oocyte maturation and also in the early embryo-specific period is essential for the normal embryo development [43,44]. Disruption of mitochondria has been observed in vitrified-warmed porcine [45] and bovine [14] oocytes. In the present study, we observed that vitrified oocytes displayed a higher percentage of oocytes showing a mitochondrial aggregate distribution. Damage to the cytoskeleton during vitrification might affect the movement of mitochondria within oocytes [46]. On the contrary, a significantly higher percentage of oocytes showing a homogeneous distribution of mitochondrion was observed in oocytes vitrified after IVM with GSH-OEt. This result reports that in vitro maturation with GSH-OEt preserve the intracellular distribution of mitochondria after vitrification. This might partially explain the observation that GSH-OEt protects against cytoskeletal injury during vitrification.

When the mitochondrial oxidative activity was assessed, similar fluorescence intensity was observed after GSH-OEt treatment in both fresh and vitrified oocytes while non-treated vitrified oocytes resulted in significantly higher activity. It has already been demonstrated that vitrification may disrupt redox status, reduce GSH content and increase both cytoplasmic and mitochondrial ROS levels, as a consequence of a higher energy requirement for the reorganization of organelles after oocyte vitrification-warming [39,47]. So, our results may indicate that in vitro maturation with GSH-OEt may have provided to the oocyte with the required levels of GSH to protect the vitrified oocytes from the oxidative stress induced by the vitrification/warming process. 

Although no beneficial effects were observed in terms of cleavage rates, oocyte IVM with GSH-OEt prior to vitrification produced embryos that were able to overcome the 8-cell barrier and develop to the D8 blastocyst stage in similar percentages as fresh non-vitrified oocytes. Mechanisms proposed for the impacts of ROS on the developmental potential of vitrified oocytes have been mitochondrial damage, ATP depletion, apoptosis, modified calcium levels during fertilization and developmental blocks [48,49]. In our study, IVM with GSH-OEt supplementation helped the embryos to develop beyond the 8-cell block. While only 25.48% of 42.84% of cleaved embryos derived from vitrified oocytes overcame this block, 37.54% of 45.50% of cleaved embryos derived from oocytes in the VIT-GSH-OEt group reached the 16-cell stage. This proportion observed in vitrified pretreated oocytes was similar to the rates recorded in the groups of fresh non-vitrified oocytes. Similar results have been reported in mouse oocytes whereby preincubation with GSH-OEt helped vitrified mouse oocytes overcome the 2-cell block [37]. Despite the protective effect of GSH-OEt being appreciable before the 8-16 cell stage, the only mechanism explaining how GSH-OEt abolished the development block is through the degradation of ROS. Oocytes IVM with GSH-OEt prior to vitrification gave rise to similar D8 blastocyst rates and hatching abilities to non-vitrified oocytes, although both percentages were not significantly different than those observed for non-treated vitrified oocytes. Improved embryo development rates derived from vitrified oocytes after IVM with GSH-OEt have been also reported in mice [30,37]. The blastocyst rates reported in this study arising both from fresh or vitrified oocytes are lower compared to data from similar studies carried out in cattle [27,50,51]. This is likely due to our use of oocytes from cows of 12 to 18 months of age. As previously observed in our laboratory [52,53], juvenile oocytes are more sensitive to freezing injury than adult oocytes due to a failure or inability of these oocytes to undergo correct nuclear and cytoplasmic maturation [54].

ROS and mitochondria play an important role in apoptosis induction. Oxidation of mitochondrial pores by ROS due to disruption of the mitochondrial membrane potential may trigger the intrinsic apoptotic pathway [17]. *BAX* is a pro-apoptotic protein that leads to cell death, whereas *BCL2* is an anti-apoptotic protein that promotes cell survival. The *BAX*:*BCL2* ratio determines whether a cell survives or undergoes apoptosis [55]. Several studies have examined how cryopreservation affects the expression of *BAX* and *BCL2* in mammalian oocytes and embryos, but results have been inconsistent. Our study revealed no differences in transcript abundances of these two genes in oocytes and their derived embryos after vitrification, in agreement with reports in sheep [56] and mice [57]. However, vitrification has been found to up-regulate both *BAX* and *BCL2* expression in bovine [58,59], porcine [60,61] and murine [37] oocytes. In a recent study, Li et al. [37] observed that *BCL2* expression was lower in vitrified murine oocytes when compared to vitrified oocytes pretreated with GHS-OEt, while the *BAX* expression level was higher in the vitrified group than in the GSH-OEt group. 

To examine how supplementation of the IVM medium with 5 mM GSH-OEt affects redox status, we analyzed two genes involved in ROS scavenging: *GPX1* and *SOD1*. Superoxide dismutase (SOD) is located in the cytoplasm and neutralizes superoxide anions (O_2_^-^) by converting them to less reactive H_2_O_2_ which can, in turn, be scavenged by glutathione peroxidase (GPX) and catalase (CAT) reactions to form H_2_O and O_2_. The addition of GSH-OEt to the IVM medium did not affect relative expression levels of *GPX1* and *SOD1* in vitrified oocytes compared to non-vitrified oocytes. According to Yan and Harding [62], the glutathione redox cycle is the protection mechanism against low grade oxidant stress, while catalase offers more protection against severe oxidant stress. This could explain why impacts on *GPX1* or *SOD1* gene expression were not apparent in our vitrified oocytes. Notwithstanding, the results of studies analyzing *GPX1* mRNA expression in fresh and vitrified/warmed oocytes have been inconsistent. Thus, the vitrification of porcine oocytes led to a significant increase in *GPX1-*transcript abundance [60], yet Sprícigo et al. [53] reported a significant decrease in *GPX1* gene expression after the vitrification/warming of bovine oocytes. As in our study, Pereira et al. [60] and Turathum et al. [61] observed similar abundances of mRNA *SOD1* transcripts in both fresh and vitrified oocytes. Although not significant, we observed higher *GPX1* expression in D8 blastocyst derived from vitrified GSH-OEt-treated oocytes. Studies in cow oocytes have shown that relative *GPX1*-transcript abundance is higher in excellent/good blastocysts compared to blastocysts classified as fair, which suggests that higher *GPX1* gene expression is associated with greater embryo quality [63].

Aquaporin 3 (AQP3) is a transmembrane channel protein that allows the rapid and passive movement of water as well as other tiny neutral solutes across the membrane to improve plasma membrane permeability and blastocyst cavity formation [64]. Thus, an analysis of *AQP3*-transcript levels in bovine blastocysts after oocyte vitrification could provide information about the permeability of the plasma membrane and the capacity for blastocyst cavity formation. Blastocysts derived from vitrified/warmed oocytes showed a significantly greater abundance of *AQP3*-transcripts than blastocysts derived from fresh oocytes. However, blastocysts derived from oocytes supplemented with GSH-OEt before vitrification showed a trend towards lower *AQP3* expression compared to those derived from non-treated vitrified oocytes. Reduced aquaporin expression has been related to increased resistance to apoptosis [65]. Accordingly, GSH-OEt supplementation before oocyte vitrification could confer in vitro produced embryos some protection against apoptosis via *AQP3* down-regulation. As no differences in *BAX* and *BCL2* gene expression levels were observed between our GSH-OEt treated and non-treated blastocysts derived from vitrified oocytes, further work is needed to confirm this hypothesis.

Levels of embryonic interferon-tau-c2 (IFN-τ) expression and secretion have been described as indicators of the developmental competence and quality of in vitro produced bovine embryos [66,67]. When levels of mRNA expression *IFN-τ* were assessed, we observed no differences in D8 blastocysts produced from fresh or vitrified/warmed in vitro matured bovine MII oocytes, regardless of prior GSH-OEt treatment. However, connexin 43 (*CX43)* gene transcripts were significantly upregulated in embryos derived from vitrified/warmed oocytes that had been in vitro matured in the presence of GSH-OEt. *CX43* expression has been related to compaction and cell-to-cell adhesion [68]. Moreover, high *CX43* expression has been related to better quality embryos and their greater cryotolerance [69].

In conclusion, the results of the present study indicate that GSH-OEt added to the IVM medium improves the cryotolerance of mature bovine oocytes to vitrification by preserving mitochondrial distribution pattern, diminishing both cytoplasmic and mitochondrial ROS levels and enhancing embryo development. No effects of GSH-OEt supplementation prior to vitrification were observed on the expression of targeted genes in oocytes and their derived blastocysts with the exception of *GPX1*, *AQP3* and *CX43* in blastocysts. These data suggest that while GSH-OEt treatment is unable to fully rescue the developmental capacity of vitrified warmed oocytes, this additional antioxidant does seem to improve the resilience of bovine in vitro matured oocytes to the oxidative stress of vitrification. 

## 4. Materials and Methods

### 4.1. Chemicals and Suppliers 

Unless otherwise specified, all chemicals and reagents used in this study were purchased from Sigma Chemical Co (St. Louis, MO, USA).

### 4.2. Oocyte Collection and In Vitro Maturation

The in vitro maturation (IVM), in vitro fertilization (IVF) and in vitro culture (IVC) protocols followed have been described elsewhere [70]. Briefly, ovaries from postpubertal heifers (12 to 18 months) were obtained from a local slaughterhouse and shipped to the laboratory in saline solution (0.9% NaCl) at 35–37 °C. Immature cumulus-oocyte complexes (COCs) were aspirated from 3–8 mm antral follicles and washed in modified Dulbecco’s PBS (PBS supplemented with 36 μg/mL pyruvate, 50 μg/mL gentamicin and 0.5 mg/mL bovine serum albumin, BSA). Only COCs with more than three compact layers of cumulus cells and a homogeneous cytoplasm were selected for IVM. Groups of up to 50 COCs were placed in 500 µL of maturation medium in four-well dishes and cultured for 24 h at 38.5°C in a 5% CO_2_ humidified air atmosphere. The maturation medium (IVM medium) consisted of tissue culture medium (TCM-199) supplemented with 10% (*v/v*) fetal bovine serum (FBS), 10 ng/mL epidermal growth factor and 50 µg/mL gentamicin. 

### 4.3. Oocyte Vitrification and Warming

In vitro matured oocytes were vitrified/warmed as previously described by Morató et al. [38].

#### 4.3.1. Vitrification Protocol

After 22 h of IVM, oocytes were partially denuded by gently pipetting in holding medium (HM: Hepes-TCM-199 supplemented with 20% (*v/v*) FBS). Oocytes with only 2–4 layers of cumulus and corona radiata cells were transferred to HM supplemented with 7.5% (*v/v*) ethylene glycol (EG) and 7.5% (*v/v*) dimethyl sulfoxide (DMSO) for 10 min and then to HM supplemented with 15% (*v/v*) DMSO, 15% (*v/v*) EG, and 0.5 M sucrose for 45 to 60 s. Up to five oocytes were loaded onto the Cryotop and almost all the solution was removed to leave only a thin layer covering the oocytes. Oocytes were immediately plunged into liquid nitrogen. The entire process from exposure to the vitrification solution to plunging in liquid nitrogen was completed within 90 s. 

#### 4.3.2. Warming Protocol

Warming was performed by quickly immersing the tip of the Cryotop in the HM supplemented with 1 M sucrose. After 1 min, oocytes were transferred into HM supplemented with 0.5 M sucrose for 3 min and then to HM for 5 min. Oocytes were then transferred back into the maturation medium and allow to mature for 2 additional hours at 38.5 °C in humidified air containing 5% CO_2_. Vitrified/warmed oocytes were assessed for viability according to their morphology under a stereomicroscope. Only vitrified/warmed oocytes showing a normal morphology (symmetrical shape and no signs of lysis) were subjected to in vitro fertilization.

### 4.4. In Vitro Fertilization and Embryo Culture

Commercially available frozen semen from an Asturian bull (ASEAVA, Llanera, Asturias, Spain) of proven fertility was used in all experimental procedures. The content of a frozen/thawed straw was layered on top of a Bovipure density gradient (1 mL 40% Bovipure on 1 mL 80% BoviPure; Nidacon Laboratories AB, Göteborg, Sweden) and centrifuged for 10 min at 300× *g*. The underlying sperm pellet was re-suspended in 3 mL of BoviWash (Nidacon International, Göteborg, Sweden) and pelleted by centrifugation at 300× *g* for 5 min. Spermatozoa were counted in a Neubauer chamber and diluted in an appropriate volume of fertilization medium (Tyrode’s medium supplemented with 25 mM bicarbonate, 22 mM Na-lactate, 1 mM Na-pyruvate, 6 mg/mL fatty acid-free BSA and 10 mg/mL heparin–sodium salt, Calbiochem, Darmstadt, Germany). Up to 50 matured oocytes were transferred to 250 µL of IVF medium and co-incubated with 250 µL of sperm suspension (final sperm concentration: 1 × 10^6^ spermatozoa/mL) at 38.5°C in a 5% CO_2_ humidified air atmosphere. 

At the time point 18–20 h post-insemination (hpi), presumptive zygotes were denuded of cumulus cells by gentle pipetting and transferred to 20 µL culture droplets (1 embryo/µL) under mineral oil. The culture medium was synthetic oviductal fluid (SOF) (Caisson Labs, Smithfield, VA, USA) supplemented with 0.96 mg/mL BSA, 88.6 mg/mL Na-pyruvate, 2% non-essential amino acids, 1% essential amino acids, 0.5% gentamicin and 5% FBS. In vitro culture was conducted at 38.5 °C in a 5% CO_2_, 5% O_2_ and 90% N_2_ humidified atmosphere. Cleavage rates were assessed at 48 h post-insemination (hpi), 16-cell stage rates at 96 hpi and blastocyst yields on Days 7 (D7) and 8 (D8) post-insemination (pi). According to IETS standards, D8 embryos were classified into three groups according to the degree of blastocoel expansion: (a) non-expanded: early blastocysts (stage code 5) and blastocysts (stage code 6); (b) expanded: expanded blastocysts (stage code 7); and (c) hatched: hatching (stage code 8) or hatched blastocysts (stage code 9).

### 4.5. Chromosome and Spindle Organization 

After 24 h of IVM, oocytes were denuded of cumulus cells by gentle pipetting and fixed in 2% (*w/v*) paraformaldehyde–PBS for 30 min. Oocytes were then permeabilized in Triton X-100 (2.5% (*v/v*) in PBS) for 20 min, blocked in 3% BSA (*w/v*) in PBS for 30 min at 38.5 °C, and immunostained for tubulin and chromatin detection as described previously by Arcarons et al. [71]. Briefly, fixed oocytes were incubated with mouse anti-α-tubulin monoclonal antibody (Molecular Probes, Paisley, UK; 1:250 dilution) overnight at 4 °C, followed by incubation with the anti-mouse IgG antibody Alexa Fluor^™^ 488 (Molecular Probes, Paisley, UK; 1:5000) at 38.5 °C for 1 h. Oocytes were washed three times in PBS at 38.5 °C for 5 min after each incubation. Groups of 20 oocytes were mounted on poly L-lysine-treated coverslips fitted with a self-adhesive reinforcement ring in a 3-μL drop of Vectashield containing 125 ng/mL 4′,6′-diamidino-2-phenylindole hydrochloride (DAPI) (Vysis Inc., Downers Grove, USA) and flattened with a coverslip. Preparations were sealed with nail varnish and stored at 4 °C protected from light until observation within the following 2 days. An epifluorescence microscope (Axioscop 40FL; Carl Zeiss, Göttingen, Germany) was used to examine tubulin (Alexa Fluor^™^ 488; excitation 488 nm) and chromatin (DAPI; excitation 405 nm). The criteria used to classify chromosome and microtubule distributions have been described elsewhere [38]. In brief, a normal meiotic spindle was defined as showing the classic symmetrical barrel shape, with chromosomes aligned regularly in a compact group along the equatorial plane. In contrast, a spindle structure was recorded as abnormal when there was microtubule decondensation or partial or total disorganization, or as absent when there was a complete lack of microtubules. Chromosome organization was considered abnormal when chromosomes were dispersed or had an aberrant, less condensed appearance or lacking when chromosomes were missing.

### 4.6. Reactive Oxygen Species

Intracellular ROS levels in oocytes were quantified after IVM by labeling with 2′,7′-dichlorodihydrofluorescein diacetate (H_2_DCFDA) following the procedure described by Castillo-Martín et al. [72] with some modifications. In short, COCs were denuded by gentle pipetting and washed twice in PBS supplemented with 1 mg/mL of polyvinyl alcohol (PVA). Oocytes with one visible polar body were then incubated in PBS-PVA supplemented with 5 μM H_2_DCFDA for 30 min at 38.5 °C in a humidified 5% CO_2_ air atmosphere. Oocytes were washed twice in PBS-PVA, placed on a slide and covered with a coverslip. Fluorescence emitted by the oocytes was captured under an inverted epifluorescence microscope (Zeiss Axio Vert.A1, Oberkochen, Germany) using a filter for 460–500 nm for excitation and 520–560 nm for emission. Fluorescence intensities were expressed in arbitrary fluorescence units (pixel) [73] using ImageJ software (Version 2.0.0-rc-69/1.52p; National Institutes of Health, Bethesda, MD, USA). Fluorescence intensities in positive control oocytes exposed to 2% hydrogen peroxide for 30 min at 38.5 °C in a humidified 5% CO_2_ air atmosphere were set at 100%, and the relative peroxide levels of the samples calculated with respect to this value.

### 4.7. Mitochondrial Activity and Distribution

The mitochondrial membrane potential was assessed using the fluorescent probe MitoTracker^®^ Red CM-H_2_XRos (MTR-CMH_2_) (M7513, Molecular Probes, Invitrogen, Eugene, OR, USA) according to the manufacturer’s instructions. MitoTracker^®^ Red CM-H_2_XRos, a reduced form of X-rosamine, does not fluoresce until it enters an actively respiring cell, where it is oxidized predominantly by ROS into the fluorescent form, and is retained in mitochondria upon depolarization [74]. MII-stage oocytes were denuded from the adherence of cumulus cells and were incubated in PBS supplemented with 500 nM dye for 30 min at 38.5 °C in a dark, humidified, 5% CO_2_ atmosphere. After incubation, oocytes were washed twice in PBS, fixed in a 2% PFA solution in PBS for 20 min at 38.5 °C and rinsed two times in PBS. Finally, nuclei were counterstained with 0.5 mg/mL of Hoechst 33342 (Molecular Probes, Invitrogen, Eugene, OR, USA) at 38.5 °C for 10 min. Groups of 20 oocytes were mounted as previously described above. Oocytes were observed in their equatorial plane using a confocal microscope Leica TCS SP5 (Leica Microsystems GmbH, Mannheim, Germany) at 405 nm for Hoescht 33342 and 561 nm for MitoTracker^®^ Red. ImageJ software (Version 2.0.0-rc-69/1.52p; National Institutes of Health, Bethesda, MD, USA) was used to quantify the fluorescence intensity of MitoTracker^®^ Red CM-H_2_XRos. Fluorescence intensity of the oocytes was measured and normalized to the average in the non-vitrified control group in each experiment. In addition, the oocytes were classified according to the distribution of the mitochondria in the cytoplasm, as described previously by Moawad et al. [75] into ‘aggregated’ or ‘non-aggregated’ according to the presence or absence of two or more aggregates in the oocyte cytoplasm. 

### 4.8. RNA Extraction, Reverse Transcription and Quantitative Real-Time PCR Analysis 

The procedures used for RNA extraction and real-time reverse transcription-quantitative polymerase chain reaction (RT-qPCR) have been described elsewhere [50]. For gene expression analysis, groups of 30 (MII) or 5 (D8 embryos) were plunged into liquid nitrogen and stored at −80 °C. Poly-(A)-RNA was extracted using the Dynabeads mRNA Direct Extraction Kit (Invitrogen™, Oslo, Norway) according to the manufacturer’s instructions with minor modifications. For poly-(A)-RNA extraction, pooled samples were lysed in 50 μL lysis buffer at room temperature for 5 min by gentle pipetting, and the fluid lysate was then hybridized with 10 mL prewashed beads, also at room temperature, for 5 min with gentle shaking. After hybridization, poly-(A)-RNA–bead complexes were washed at room temperature twice in 50 μL Washing Buffer A and two further times in 50 μL Washing Buffer B. Next, samples were eluted in 16 μL elution buffer (Tris-HCl) and heated to 70 °C for 5 min. Immediately after extraction, 4 μL qScript cDNAsupermix (Quanta Biosciences; Gaithersburg, MD, USA) was added and reverse transcription (RT) was performed using oligo-dT primers, random primers, dNTPs and qScript reverse transcriptase. The RT reaction was run for 5 min at 25 °C, followed by 1 h at 42 °C to allow the RT-qPCR of mRNA, and 10 min at 70 °C to denature the reverse transcriptase enzyme. After RT, the resulting cDNA was diluted in 25 μL Tris-HCl (elution solution). 

The relative abundance of mRNA transcripts was quantified by qPCR using a 7500 Real-Time PCR System (Applied Biosystems, Foster City, CA, USA). The qPCR mix contained 10 μL Fast SYBR Green Master Mix (Applied Biosystems, Foster City, CA, USA), 1.2 μL each primer (300 nM; Life Technologies, Madrid, Spain) and 2 μL cDNA template. Nuclease-free water was added to make up a final volume of 20 μL. The PCR amplification consisted of one cycle of denaturation at 95 °C for 10 min, followed by 45 cycles of amplification with a denaturation step at 95 °C for 15 s, annealing step for 1 min at 60 °C (the appropriate annealing temperature for the primers) and a final extension step at 72 °C for 40 s. Fluorescence data were acquired during the final extension step. The identity of the amplified PCR products was verified by melting curve analysis and gel electrophoresis (on a 2% agarose gel containing 0.1 μg/mL SafeView; Applied Biological Materials, Vancouver, Canada). The melting protocol consisted of heating the samples from 50 to 95 °C and holding at each temperature for 5 s while monitoring fluorescence. In each run, there were three technical replicates from each of the three biological replicates per individual gene. Negative controls for the template and for the RT were also included and amplified by PCR to ensure no cross-contamination. 

Five candidate genes (*BAX*, *BCL2*, *GPX1*, *SOD1* and *AQP3*) for MII oocytes and seven genes (*BAX*, *BCL2*, *GPX1*, *SOD1*, *AQP3*, *IFN-τ* and *CX43*) for D8 blastocysts were used to perform quantitative PCR analysis in comparison with endogenous control genes (peptidylprolyl isomerase A, *PPIA*; and H3 histone, family 3A, *H3F3A*). The comparative threshold cycle (ΔΔ*C*t) method [76] was used to quantify relative gene expression levels and quantification was normalized to the endogenous control (housekeeping (HK) genes: *PPIA* and *H3F3A)*. Fluorescence data were acquired after each elongation step to determine the threshold cycle for each sample. The threshold cycle, which is set on the log-linear phase, reflects the PCR cycle number at which the fluorescence generated within a given reaction is just above background fluorescence. Within this region of the amplification curve, a difference of one cycle is equivalent to doubling of the amplified PCR product. According to the comparative Ct method, the Δ*C*t value was determined by subtracting the mean between *PPIA* and *H3F3A* Ct values for each sample from the Ct value of each target gene of the sample for each replicate separately. Calculation of ΔΔ*C*t involved the subtraction of the Δ*C*t value for the fresh oocyte control group from all the other Δ*C*t sample values. Fold differences in relative transcript abundances were calculated for target genes assuming an amplification efficiency of 100% using the formula 2^−ΔΔ*C*t^, or Livak and Schmittgen method [77]. Primer sequences, amplicon size and GenBank accession numbers for each gene are provided in Table 3. The efficiency of primer amplification was 100%. Non-template controls were not amplified or returned a *C*t value 10 points higher than the average *C*t value for all genes. The experiment was repeated independently three times.

### 4.9. Experimental Design 

#### 4.9.1. Meiotic Spindle Status, ROS Production, Mitochondrial Activity and Distribution and Gene Expression Examined in Vitrified/Warmed Bovine Oocytes after Maturation in IVM Medium with or without GSH-OEt

After collection, COCs were randomly assigned to two IVM groups: (1) Control: oocytes in vitro matured in IVM medium and (2) GSH-OEt: oocytes in vitro matured in IVM medium supplemented with 5 mM GSH-OEt. This GSH-OEt concentration was chosen based on the findings of a previous study [27]. After 22 h of IVM, half of the oocytes in each IVM group were vitrified/warmed using the Cryotop method to give rise to the vitrification groups VIT Control and VIT GSH-OEt and allowed to recover in their respective IVM media for two additional hours. After 24 h of IVM, a sample of oocytes from each of the four treatment groups (Control, GSH-OEt, VIT Control and VIT GSH-OEt) was collected to assess spindle and chromosome configurations (three replicates), ROS production (three replicates) or mitochondrial oxidative activity and distribution (three replicates). For gene expression, a pool of oocytes from each group was denuded of cumulus cells by gentle pipetting and 30 oocytes showing extrusion of the first polar body were collected from each treatment group, snap-frozen in liquid nitrogen and stored at −80°C until RNA extraction and RT-qPCR analysis (four replicates). 

#### 4.9.2. Developmental Competence and Gene Expression Determined in Embryos Derived from Vitrified/Warmed Bovine Oocytes In Vitro Matured in IVM Medium with or without GSH-OEt

After collection, COCs were randomly assigned to two IVM groups: (1) Control: oocytes in vitro matured in IVM medium and (2) GSH-OEt: oocytes in vitro matured in IVM medium supplemented with 5 mM GSH-OEt. After 22 h of IVM, half of the oocytes in each IVM group were vitrified/warmed using the Cryotop (VIT Control and VIT GSH-OEt) and allowed to recover in their respective IVM media for two additional hours. After 24 h of IVM, oocytes in each of the treatment groups (Control, GSH-OEt, VIT Control and VIT GSH-OEt) were inseminated and in vitro cultured for 8 days. Cleavage rates, 16-cell stage embryos and blastocysts were assessed at 48 hpi, 96 hpi and D7 and D8 pi, respectively. In each group, D8 embryos were classified as blastocysts (stage code 6), expanded (stage code 7), or hatching/hatched (stage code 8 and 9), pooled in groups of 5, snap-frozen in liquid nitrogen and stored at −80 °C until RNA extraction and RT-qPCR analysis (three replicates per group).

## 5. Statistical Analyses

Data were analyzed using statistical package R, Version R 3.4.4. The normality of data distribution was checked using the Shapiro–Wilk test and homogeneity of variances through the Levene test. When required, data were linearly transformed into √x, arcsin √x or log(x) prior to running statistical tests. A linear mixed-effect followed by a pairwise comparison test (Tukey-adjustment) was used to assess differences between groups in spindle configuration, ROS production, mitochondrial oxidative activity and embryo development. The nonparametric Kruskal–Wallis test was used to examine the percentage in mitochondrial distribution and relative transcript abundances differences among the treatment groups. The data from the different groups were compared using the nonparametric Mann–Whitney U test. Significance was set at *p ≤* 0.05.

## Figures and Tables

**Table 1:** Spindle configurations observed in MII bovine oocytes in vitro matured in in vitro maturation (IVM) medium with or without glutathione ethyl ester (GSH-OEt) before their vitrification.

	*N*	MII (%)	Normal	Microtubule Configuration (%) *		Chromosome Distribution (%) *	
				Abnormal	Absent	Abnormal	Absent
**Control**	127	79.4 ± 9.2	74.1 ± 4.4	16.2 ± 5.6 ^a^	9.7 ± 1.1	25.9 ± 8.3	0
**GSH-OEt**	113	76.5 ± 6.5	65.6 ± 5.7	26.2 ± 9.0 ^ab^	8.1 ± 4.3	33.2 ± 10.6	1.1 ± 1.1
**VIT Control**	71	60.0 ± 5.6	54.8 ± 7.8	32.7 ± 10.8 ^b^	12.4 ± 1.4	45.2 ± 14.5	0
**VIT GSH-OEt**	53	57.4 ± 10.7	58.5 ± 8.8	31.1 ± 9.3 ^ab^	10.4 ± 5.8	41.5 ± 14	0

^a,b^ Different letters indicate significant differences (*p* < 0.05). * Percentage referred to the total number of oocytes at MII. Data shown as mean ± SEM. Treatment groups: *Control*, oocytes in vitro matured in IVM medium; *GSH-OEt*, oocytes in vitro matured in IVM medium supplemented with 5 mM GSH-OEt; *VIT Control*, oocytes in vitro matured in IVM medium and then vitrified on Cryotops followed by warming; *VIT GSH-OEt*, oocytes in vitro matured in IVM medium supplemented with 5 mM of GSH-OEt and then vitrified on Cryotops followed by warming.

**Table 2:** Developmental competence of embryos derived from bovine oocytes vitrified/warmed after their maturation in IVM medium supplemented with GSH-OEt.

	*n*	Cleavage Rate 48 hpi	16-Cell Embryo 96 hpi	Blastocyst Yields		D8 Blastocysts			
				D7 Blastocyst	D8 Blastocyst	n_D8_	Non-Expanded	Expanded	Hatched
Control	381	73.09 ± 3.84 ^a^	53.74 ± 8.80 ^a^	14.74 ± 1.71 ^ab^	23.12 ± 5.17 ^a^	85	48.04 ± 14.66	24.85 ± 2.76	27.11 ± 14.70 ^a^
GSH-OEt	307	74.76 ± 3.26 ^a^	45.62 ± 2.95 ^a^	20.18 ± 6.45 ^a^	28.59 ± 8.06 ^a^	84	35.86 ± 9.95	41.48 ± 5.48	22.66 ± 5.40 ^a^
VIT Control	136	42.84 ± 6.88 ^b^	25.48 ± 4.10 ^b^	4.09 ± 1.30 ^b^	5.26 ± 0.58 ^b^	7	44.44 ± 29.40	55.56 ± 29.40	0 ^b^
VIT GSH-OEt	134	45.50 ± 10.16 ^b^	37.54 ± 2.44 ^ab^	6.62 ± 0.81 ^b^	13.78 ± 3.20 ^ab^	20	50.43 ± 12.75	40.04 ± 6.71	9.52 ± 9.52 ^ab^

^a,b^ Different letters indicate significant differences (*p < 0.05*). * Day 7 and Day 8 blastocyst yields were calculated as proportions of the total number of oocytes inseminated at 24 hpi (*n*). Data are shown as mean ± SEM. *Control*, oocytes in vitro matured in IVM medium; *GSH-OEt*, oocytes in vitro matured in IVM medium supplemented with 5 mM GSH-OEt; *VIT Control*, oocytes in vitro matured in IVM medium and then vitrified on Cryotops followed by warming; *VIT GSH-OEt*, oocytes in vitro matured in IVM medium supplemented with 5 mM of GSH-OEt and then vitrified on Cryotops followed by warming.

**Table 3:** Primer sequences used for RT-qPCR relative gene expression analysis.

Symbol	GenBank Accession Number	Primer Sequence (5′–3′)	Fragment Size (bp)
*BAX*	NM_173894.1	F: ACCAAGAAGCTGAGCGAGTG	116
		R: CGGAAAAAGACCTCTCGGGG	
*BCL2*	NM_001166486.1	F: GAGTTCGGAGGGGTCATGTG	211
		R: TGAGCAGTGCCTTCAGAGAC	
*GPX1*	NM_174076.3	F: CTGAAGTACGTCCGACCAGG	153
		R: GTCGGTCATGAGAGCAGTGG	
*SOD1*	NM_174615.2	F: ACACAAGGCTGTACCAGTGC	102
		R: CACATTGCCCAGGTCTCCAA	
*AQP3*	NM_001079794.1	F: GTGGACCCCTACAACAACCC	222
		R: CAGGAGCGGAGAGACAATGG	
*IFN-τ*	AF238612	F: CTGAAGGTTCACCCAGACCC	197
		R: GAGTCTGTTCATTCGGGCCA	
*CX43*	NM_174068.2	F: TGGAATGCAAGAGAGGTTGAAAGAGG	294
		R: AACACTCTCCAGAACACATGATCG	
*PPIA*	NM_178320.2	F: CATACAGGTCCTGGCATCTTGTCC	108
		R: CACGTGCTTGCCATCCAACC	
*H3F3A*	NM_001014389.2	F: CATGGCTCGTACAAAGCAGA	136
		R: ACCAGGCCTGTAACGATGAG	

Abbreviations: *BAX*, BCL2 associated X, apoptosis regulator; *BCL2*, BCL2 apoptosis regulator; *GPX1*, glutathione peroxidase 1; *SOD1*, superoxide dismutase 1; *AQP3*, aquaporin 3; *IFN-τ*, interferon-tau-c2; *CX43*, connexin 43. *PPIA*, peptidylprolyl isomerase A, and *H3F3A*, H3 histone, family 3A (H3-3B), served as housekeeping genes.
